# Bioinspired Collagen/Gelatin Nanopillared Films as a Potential Implant Coating Material

**DOI:** 10.1021/acsabm.2c00633

**Published:** 2022-10-07

**Authors:** Pinar Alpaslan Erturk, Sevde Altuntas, Gulseren Irmak, Fatih Buyukserin

**Affiliations:** † TOBB University of Economics and Technology, Biomedical Engineering, 06560Ankara, Turkey; ‡ University of Health Sciences Turkey, Tissue Engineering Department, Experimental Medicine Research and Application Center, Validebag Research Park, 34662Istanbul, Turkey; § Malatya Turgut Ozal University, Department of Bioengineering, 44210Malatya, Turkey

**Keywords:** Collagen-gelatin nanopillar, Sharpey’s Fibers, biomimetic, osteogenic differentiation, implant coating, anodized alumina

## Abstract

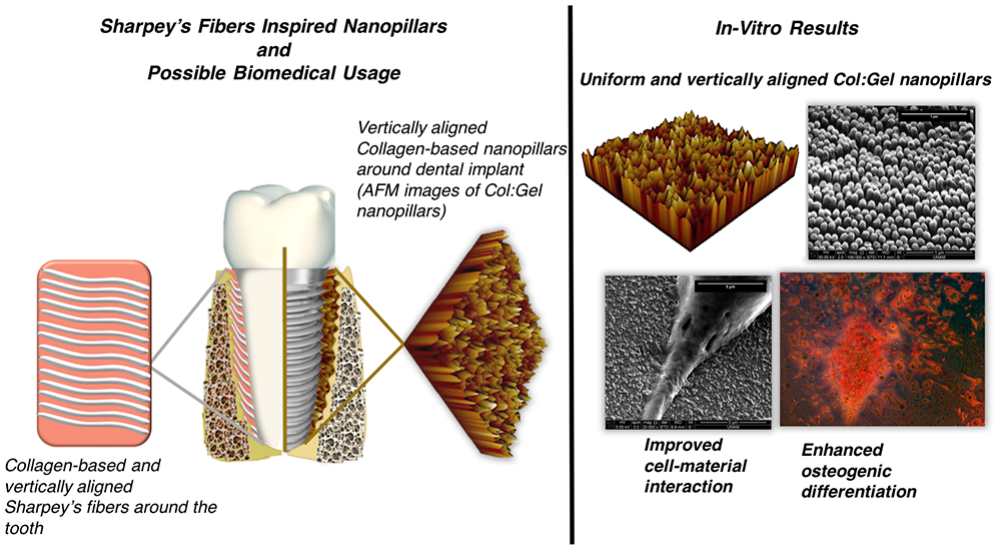

Collagen-based Sharpey’s fibers are naturally located between alveolar bone and tooth, and they have critical roles in a well-functioning tooth such as mechanical stability, facile differentiation, and disease protection. The success of Sharpey’s fibers in these important roles is due to their unique location, vertical alignment with respect to tooth surface, as well as their micronanofiber architecture. Inspired by these structures, herein, we introduce the use of nanoporous anodic aluminum oxide molds in a drop-casting setup to fabricate biopolymeric films possessing arrays of uniform Collagen:Gelatin (Col:Gel) nanopillars. Obtained structures have diameters of ∼90 nm and heights of ∼300 nm, yielding significantly higher surface roughness values compared to their flat counterparts. More importantly, the nanostructures were parallel to each other but perpendicular to the underlying film surface imitating the natural collagenous structures of Sharpey’s fibers regarding nanoscale morphology, geometrical orientation, as well as biochemical content. Viability testing showed that the nanopillared Col:Gel films have high cell viabilities (over 90%), and they display significantly improved attachment (*ca*. ∼ 2 times) and mineralization for Saos-2 cells when compared to flat Col:Gel films and Tissue Culture Polystyrene (TCPS) controls, plausibly due to their largely increased surface roughness and area. Hence, such Sharpey’s fiber-inspired bioactive nanopillared Col:Gel films can be used as a dental implant coating material or tissue engineering platform with enhanced cellular and osteogenic properties.

## 1 Introduction

Dental implants are utilized to replace a lost tooth that may occur due to an injury, tooth decay, periodontal disease, or other reasons.^1^ The long-term success of dental implant procedures depends on two important parameters: The first is osseointegration, which occurs at the bone-implant interface and is defined as “the close contact of bone and implant”.^2^ The second is the tight soft tissue integration^3^ of the implant that occurs at the implant-soft tissue interface (transmucosal part).^4^ Hence, poor integration of the implant with bone or soft tissue can cause delayed wound healing via local infection and/or implant detachment. Therefore, researchers have focused on enhancing osseointegration by a variety of routes involving physical and chemical surface modifications as well as coating the surface with polymeric and ceramic biomaterials.^5,6^ Among these modifications, coating the implant surface with biomimetic polymeric materials can promote the creation of an extracellular matrix (ECM)-like environment to enhance the cellular attachment and other biological activities of the cells such as mineralization.

Collagen is the main component of ECM of all oral tissues interacting with the dental implant, such as the cementum, which is a calcified tissue covering the root of the tooth, alveolar bone, and periodontal ligament, which is a dense fibrous connective tissue located between the cementum and alveolar bone.^7^ Collagen is responsible for cellular attachment, mechanical improvement, and mineralization of ECM, due partly to its integrin receptors which include RGD (arginine, glycine, aspartic acid) and GFOGER sequences.^8,9^ In addition, it regulates bone hemostasis and immunogenicity mainly through its hydrophilic nature and RGD-rich content.^9−12^

Collagen is found as a fibrillary (micronano scale) form ordered in a specific arrangement to satisfy the appropriate mechanical requirements and to provide a suitable differentiation environment for cells.^12^ These collagen fibers, also called Sharpey’s fibers, have diameters in the micro/nano scale, are located between alveolar bone and tooth, and run perpendicular to the tooth surface.^10^ This unique location allows them to form a mechanical and physiological transition between alveolar bone and cementum. Sharpey’s fibers not only act as a mechanical bridge between bone and tooth but also provide a favorable ECM environment for cells. Furthermore, Sharpey’s fibers, with one end in bone and the other in cement, are partially or completely mineralized.^10,13^

An interesting feature of Sharpey’s fibers is their vertical alignment in the cementum, periodontal ligament, and alveolar bone, which implies that their orientation is perpendicular to the tooth surface but the fibers are parallel to each other. The vertical orientation of the fibers is necessary to defend the bone tissue against the external stimulus, to create a strong and stable connection, and to seal soft tissue.^3,14^ Studies have shown that the vertical orientation of collagen fibers is more effective than the parallel orientation against infections that may occur at the bone-implant interface.^3,15^ Efforts to mimic the composition and orientation of these collagenous fibers are generally conducted via electrospinning, which can produce nanofibers that align parallel to each other but cannot have the vertical orientation. Even in that form, improved osteogenic characteristics have been reported elsewhere in the literature.^16,17^ Thus, a notable bioinspired design for further improvement of osseointegration can be possible by mimicking the sophisticated architecture of Sharpey’s collagenous fibers to augment cell adhesion and differentiation in the osteogenic direction.

Nanoporous anodic aluminum oxide membranes (AAOMs) are unique materials that present several advantages for the fabrication of such vertically aligned nanoscale collagen fibers. Application of appropriate anodization conditions yield membranes that contain nanopore arrays with hexagonal honeycomb arrangement. It is also possible to fine-tune the pore dimensions, which are aligned parallel to each other but perpendicular to the underlying aluminum substrate. After a facile pore surface modification, they can be utilized as molds to fabricate arrays of ordered nanopillars from both synthetic and biologic polymers for applications that span solar cells^18^ to functional biomaterials.^19^ For instance, we have recently reported the use of such molds to produce chitosan-based nanopillared films that present superb antibacterial properties and at the same time can induce osteoblastic differentiation pathways yielding significant mineralization capabilities.^19^ It was concluded that compared to flat films of the same composition, nanostructed films not only mimic the morphology of the natural ECM components but also allow higher levels of protein adsorption and focal adhesion sites for cells that yields significantly higher osteogenic outputs.^19^

In this study, we introduce a Sharpey’s fibers mimetic biomaterial possessing both the geometrical nanoarchitecture and composition of these natural fibers as a potential implant coating surface that can enhance adhesion, proliferation, and mineralization of cells, indicating evidence of early osseointegration. To the best of our knowledge there has been no report in the literature that can mimic the specific geometrical conformation and collagenous compositional characteristics of Sharpey’s fibers for such purposes. Here in, AAOM molds were utilized in a drop casting setup to fabricate films having vertically aligned nanopillar arrays composed of collagen and gelatin. Gelatin was added as a filler material to improve film processability as well as to increase the stability of collagen, since gelatin interacts with collagen fibers and fills the gap between them.^20^ Gelatin obtained as a result of the denaturation of collagen has all the biological and physical properties of collagen, as well as being advantageous over collagen due to it is nonallergenic properties and water solubility.^21^ Collagen and gelatin were cross-linked via poly(ethylene glycol) diglycidyl ether (PEGDE). Collagen/gelatin (Col:Gel) nanopillared films formed by using AAOM molds were characterized in terms of chemical content, surface morphology/roughness, and degradation profiles. The attachment, viability, and mineralization of Saos-2 cells were then analyzed with cell culture studies, where Tissue Culture Polystyrene (TCPS) and flat Col:Gel films were used as controls. The results support our hypothesis; Sharpey’s fiber-mimetic Col:Gel nanopillar films can enhance osseointegration as an implant coating material.

## 2 Materials and Methods

### 2.1 Materials

Pure grade acetone, hexane, PEGDE, NaOH, H_3_PO_4_, H_2_SO_4_, C_2_H_2_O_4_ (oxalic acid), β-glycerophosphate, Trypsin-EDTA, Trypan blue, Alizarin Red S, Penicillin–streptomycin, Fetal calf serum, DMEM, and ALP kit were purchased from Sigma-Aldrich. High purity Al foils (99.999%, Puratronic, 1 mm thickness) were obtained from Alfa Aesar, and Si ⟨111⟩ wafer was obtained from Micro Chemicals GmbH. Collagen and Gelatin were purchased from NeoCell Super Collagen and Halavet Food, Industry and Trade Inc., respectively. Micro BCA protein kit and DAPI were obtained from Thermo Scientific. ProteinEX was received from Gene All; PBS (10×) was purchased from Amresco; l-glutamine was obtained from Gibco; WST-1 kit was purchased from Cayman Chemical.

### 2.2 Nanoporous Anodic Aluminum Oxide Membrane (AAOM) Synthesis

The two-step anodization method was used to produce nanoporous AAOMs.^22,23^ Briefly, ultrapure aluminum foils (99.99%) were mechanically polished with 600 grit sandpaper and cleaned with purified water and acetone. Then, they were subjected to electropolishing in a mixture of 95 wt % H_3_PO_4_, 5 wt % H_2_SO_4_, and 20 g/L of CrO_3_ against a Pb cathode at 65 °C under 15 V for 60 min. The first anodization was performed in a 0.3 M aqueous oxalic acid solution as an electrolyte against stainless steel for at least 8 h at 5 °C under 50 V. The formed irregular alumina layer was removed in an aqueous solution composed of 0.2 M CrO_3_ and 0.4 M H_3_PO_4_ at 75 °C. Then, second anodization was performed using the same electrolyte solution for 163 s at 5 °C under 50 V. Afterward, the membranes were treated with 5 vol % H_3_PO_4_ solution for 52 min for pore widening.

Finally, the prepared membranes were coated with hydrophobic octadecyl trichlorosilane (ODTS) to reduce surface energy. For the coating process, AAOMs were incubated in 0.005% (v/v, in hexane) ODTS solution overnight. The silane-treated nanoporous AAOMs were then dried at 90 °C for 4 h until the fabrication of nanostructured films.

### 2.3 Fabrication of Nanopillared Collagen/Gelatin Films

The produced AAOM substrates were used as molds for the fabrication of collagen/gelatin (Col:Gel) nanopillar films with ordered nanopillar arrays. A Col:Gel solution (2%, w/v) was prepared by mixing collagen and gelatin (1/3, w/w) in deionized water. Then, 0.3% (v/v) poly(ethylene glycol) diglycidyl ether (PEGDE) as a cross-linker was added to the Col:Gel solution, and the pH of the solution was adjusted to 6.5 to increase the efficiency of PEGDE cross-linking.^19,24^ The Col:Gel solution was drop-cast on hydrophobically modified AAOM molds and dried at room temperature. Finally, the films were peeled from the substrates to obtain the nanopillared Col:Gel films. Flat Col:Gel films obtained from flat ODTS-modified Si wafer ⟨111⟩ were used as a control group to examine the effect of the nanotopography.

### 2.4 Characterization Studies

#### 2.4.1 Morphological Characterization of AAOMs and Col:Gel Films

##### Environmental Scanning Electron Microscopy (ESEM)

ESEM (FEI, Quanta 200, 30 kV accelerating voltage) was used for the morphological characterization of AAOMs and Col:Gel films. Before SEM imaging, samples were coated with an Au–Pd precision etching coating system. The sizes of the AAOM nanopores and Col:Gel nanopillars were calculated via ImageJ software by using the micrographs.

##### Atomic Force Microscopy (AFM)

AFM (EZ-AFM, NanoMagnetics Instruments) was utilized to measure the roughness of nanopillared and flat polymer films and to investigate the 3D structure of Col:Gel films.

#### 2.4.2 Chemical Characterization of Col:Gel Films

Fourier transform infrared spectroscopy (FTIR, Mattson 1000) was conducted for the chemical analysis of cross-linked and non-cross-linked Col:Gel films and also to confirm the existence of cross-linker in the film over a range between 600 and 2300 cm^–1^. The results were interpreted by using the Origin Software.

#### 2.4.3 Mechanical Characterization Col:Gel Films

Tensile strength and elongation at break of the Col:Gel films were measured by Instron 5944 universal material tensile testing machine. The specimens were prepared as rectangular strips having 8.5 cm length and 4 cm width which were tested at a stretching speed of 0.5 mm/min.

#### 2.4.4 Swelling and Degradation Studies of Col:Gel Films

Swelling and degradation behavior of Col:Gel films were determined by using gravimetric measurements. For swelling studies, Col:Gel films were weighed (*W*_i_) and immersed in phosphate buffer saline (PBS, pH:7.4) at 37 °C. Swollen films were taken out from the medium and weighed (*W*_f_) after wiping the excess water with a filter paper at determined time points (1, 6, 24, 48, 72, 96, 120, and 144 h).^25^ The swelling ratio was calculated according to eq 1.
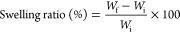
1

The degradation study of Col:Gel films was performed by incubating in phosphate buffer saline PBS, (pH 7.4) at 37 °C for different time intervals (days 1, 3, 7, and 10).^26^ Films were weighed before they were immersed in PBS and then marked as *W*_i_. At determined time points, the films were rinsed with fresh water and then dried at 50 °C under a vacuum to a constant weight prior to measurement and marked as *W*_f_. The remaining weight percentage of films was calculated using eq 2:
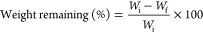
2Besides, Col:Gel films were soaked in DMEM for 3 and 24 h to analyze the nanopillar stability in the cell culture medium to mimic the cellular environment.

### 2.5 Cell Culture Studies

Cell culture studies were carried out with the Saos-2 cell line (human osteosarcoma cell line, ATCC HTB-85). The cells were cultured in a growth medium of Dulbecco’s modified Eagle’s medium F-12 (DMEM/F-12) supplemented with 10% fetal calf serum (FCS), 1% (v/v) penicillin/streptomycin, and 1% (v/v) l-glutamine in a CO_2_ incubator at 37 °C and 5% CO_2_.

The cell culture studies were conducted with three groups: (1) TCPS surface as a control group, (2) flat Col:Gel films, and (3) nanopillared Col:Gel films. Before the experiments, Col:Gel films were sterilized using 70% ethanol solution and UV sterilizer at 254 nm. Then, cells were seeded on films and TCPS surfaces and incubated in a CO_2_ incubator in a growth medium. After 24 h, the medium was replaced with an osteogenic medium which was supplemented with 10 mM β-glycerophosphate, 50 μg/mL ascorbic acid, and 10 nM dexamethasone, and the osteogenic cell medium was replaced twice a week.^27^

#### 2.5.1 Cell Viability

For the viability analysis, cells were seeded at a density of 2.5 × 10^4^ cells per well, and culture was performed on the 48 well-plates. The viability of Saos-2 cells on the films and TCPS surface were examined via WST-1 assay on the second day after seeding. Briefly, 10 μL of WST-1 solution was added to the culture medium and incubated for 2 h. Then, the cell medium was transferred to a 96-well plate and the absorbance values were read at 450 nm using a microplate spectrophotometer (Thermo Scientific Multiskan GO).

#### 2.5.2 Cell Adhesion with DAPI Staining

Cell adhesion was investigated via 4′,6-diamidino-2-phenylindole (DAPI) staining. Briefly, films seeded with Saos-2 cells at a density of 1.8 × 10^4^ cells/well were incubated on a 24-well plate. After 2 days of incubation, the medium was removed, and cells were fixed in glutaraldehyde 4% (v/v) for 30 min. After that, cells were incubated with DAPI (5 mg/mL) for nucleus staining for over 30 min. The dye was then removed, and samples were washed with PBS three times. Lastly, samples were visualized under a fluorescence microscope (Leica DMI3000 B). Images were taken from different regions of films, and the number of cells (nuclei) per unit area was calculated.

#### 2.5.3 SEM Analysis

Attachment of Saos-2 cells onto the nanopillared film surface was also observed by SEM. Cells were fixed with glutaraldehyde (4%, v/v) for 30 min on the 21st day of culture. Then the cells were washed with PBS and were dehydrated in ethanol series (20, 40, 60, and 80%, v/v) for 2 min. Lastly, the cells were treated with 98% ethanol for 1 h and were lysed. Before the SEM imaging, samples were coated with a gold–palladium layer. Quantitative analysis of calcium nodules was performed by energy-dispersive X-ray spectroscopy (EDAX).^28^

#### 2.5.4 Determination of Mineralization by Alizarin Red Staining

In order to observe the calcium deposition and mineralized nodules of the Saos-2 cells on the films and TCPS surface, Alizarin Red S staining was carried out^29^ on the 21st day of culture. In this study, cells were seeded at a density of 4 × 10^4^ cells/well, and culture was performed on the 48-well plate for 21 days.^30^ The culture medium was refreshed with the osteogenic medium twice a week. Before the staining, the cells were fixed in the glutaraldehyde (4%, v/v) for 30 min and then were washed 3 times with sterile distilled water. After that, samples were incubated with Alizarin Red S (40 mM, pH:4.2) for 30 min in dark conditions. Finally, samples were washed with sterile distilled water after staining, and they were visualized on an inverted optical microscope (Leica DMIL LED).

### 2.6 Statistical Analysis

All quantitative values are represented as means±, and all experiments were performed in triplicate for each group. Student’s *t* test was used to determine differences between groups, and a *p*-value of less than 0.05 was considered statistically significant.

## 3 Results and Discussion

### 3.1 Characterization of AAOM Molds

**Figure 1:**
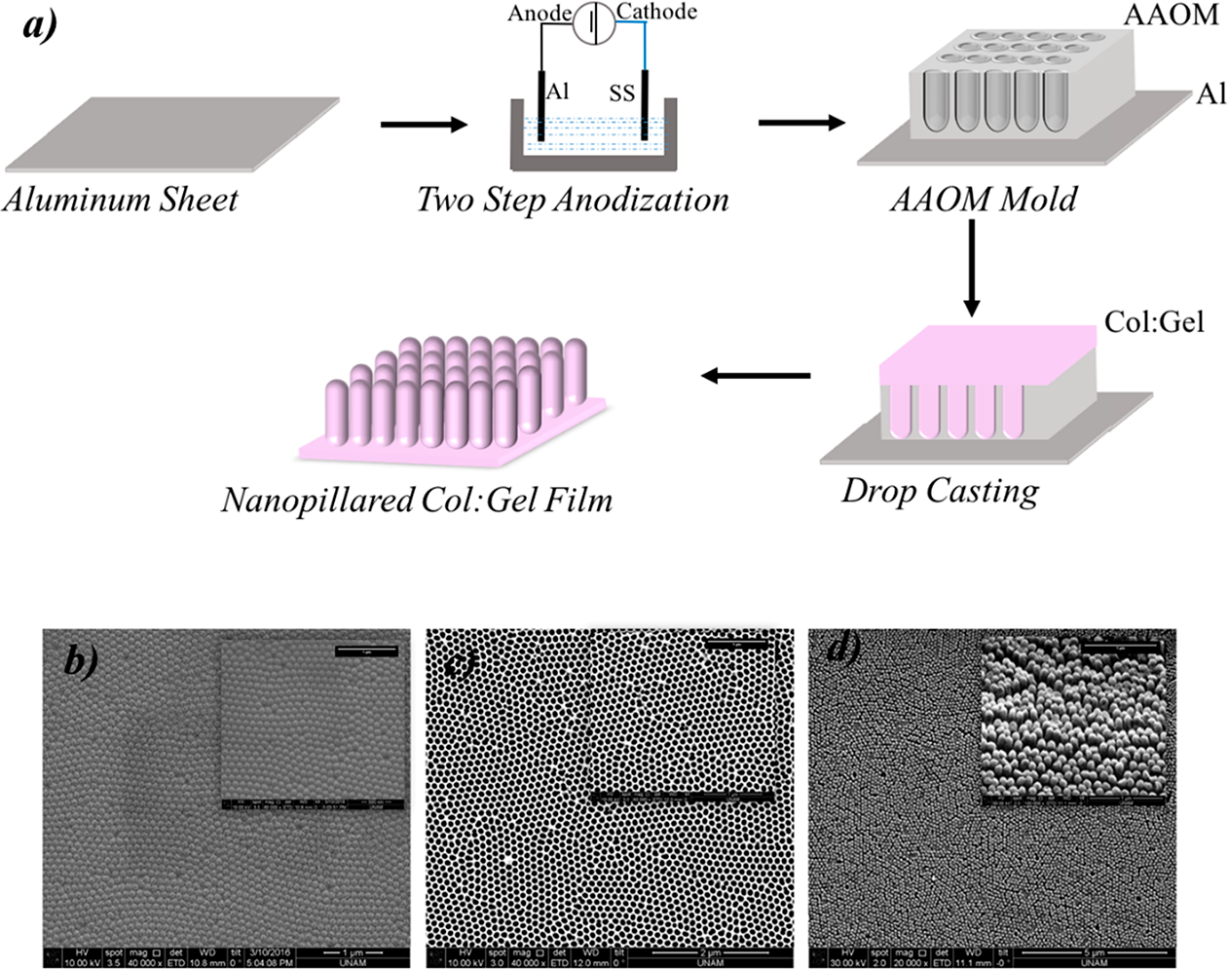
(a) Schematic diagram of manufacturing processes of AAOM mold and nanopillared Col:Gel film. (b,c) SEM images of produced AAOM: (b) before pore widening treatment, (c) after pore widening treatment with phosphoric acid (20,000×). (d) SEM image of Col:Gel nanopillar film manufactured from AAOM mold (20,000×) (Insets: (100,000×).

AAOMs were successfully synthesized using the two-step anodization method according to previous reports.^19,22^ A schematic illustration of the fabrication of AAOMs is given in Figure 1a. Morphological characterization of the molds before and after H_3_PO_4_ treatment are given in Figure 1b and c, respectively. The pore widening step with this acid clearly reveals the open pores of the membranes that form arrays of homogeneous cylindrical openings. Results of SEM analysis showed that uniform and nanoscale pores with diameters of 104.107 ± 2.34 nm were created on the AAOMS (Figure 1c) via two-step anodization confirming our previous studies.^19,31^

### 3.2 Fabrication and Characterization of Col:Gel Films

Col:Gel nanopillar structures were successfully fabricated by the drop-casting technique (Figure 1a) using the AAOM molds. We confirmed the morphologies of nanopillared films through SEM and AFM analysis. As seen in Figure 1d, the Col:Gel films consist of closely packed and well-ordered individual nanopillars with an interpillar spacing of ∼50 nm, diameters of ∼90 nm, and heights of ∼300 nm.

The Sharpey’s fibers, which we were inspired by morphology and composition, have diameters varying in the submicron to micron scale.^13,32,33^ Studies also showed that Sharpey’s fibers have a similar trend in terms of orientation (vertically) and density in alveolar bone and cementum.^10^ Within the scope of this study, submicron and vertically aligned collagen-based nanopillars were formed in a similar configuration with the native Sharpey’s fibers. The effect of vertically ordered and nanosized pillars on cell–material interaction will be discussed in the cell culture section.

**Figure 2:**
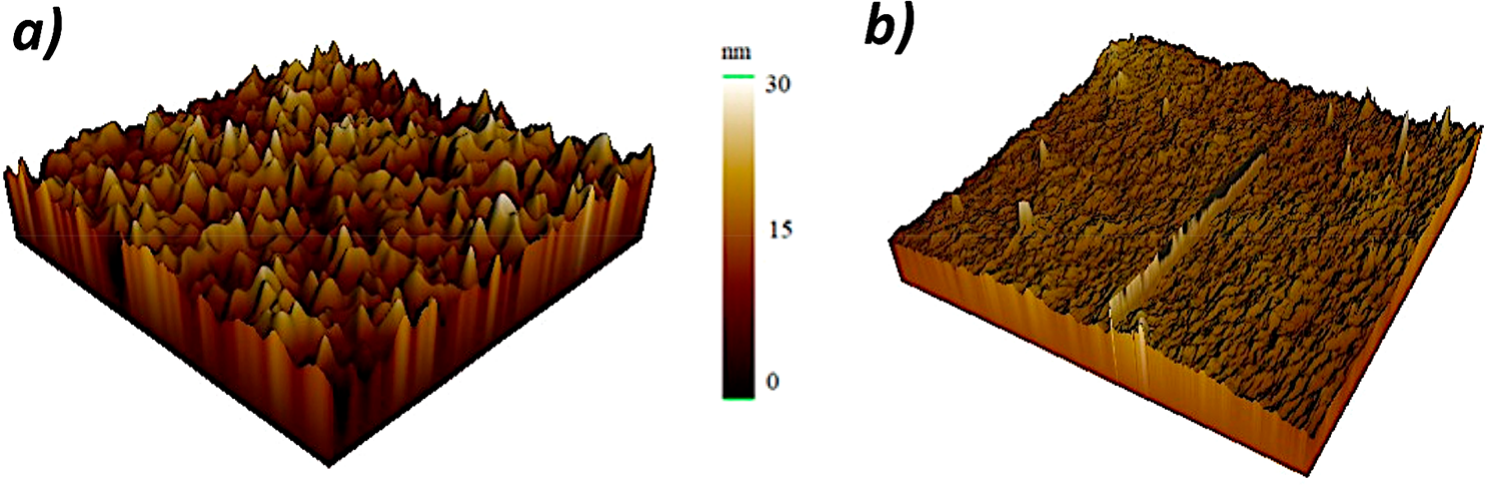
Three-dimensional images of Col:Gel films acquired by AFM: (a) nanopillar Col:Gel film and (b) flat Col:Gel film.

To evaluate the effect of nanopillar architecture on cellular behavior, flat Col:Gel films were formed using silicon wafers and used as a control group. Both nanopillared and flat Col:Gel films were characterized by AFM (Figure 2), and the differences between the nanopillared and flat film surfaces were identified by the roughness values (Table 1).

**Table 1:** Surface Roughness Parameters of Col:Gel Films Obtained via AFM

Col:Gel films	Surface roughness values (μm)
Nanopillared film	0.350
Flat film	0.030

According to the results, the surface roughness value of nanostructured films is significantly higher than that of flat Col:Gel films (Figure 2, Table 1). Order of magnitude higher roughness values for films obtained from the nanoporous mold were expected when compared to the atomically smooth Si substrate utilized for the flat counterpart. The influence of high roughness value on the cell–material interaction was researched and will be discussed in the cell culture section.

**Figure 3:**
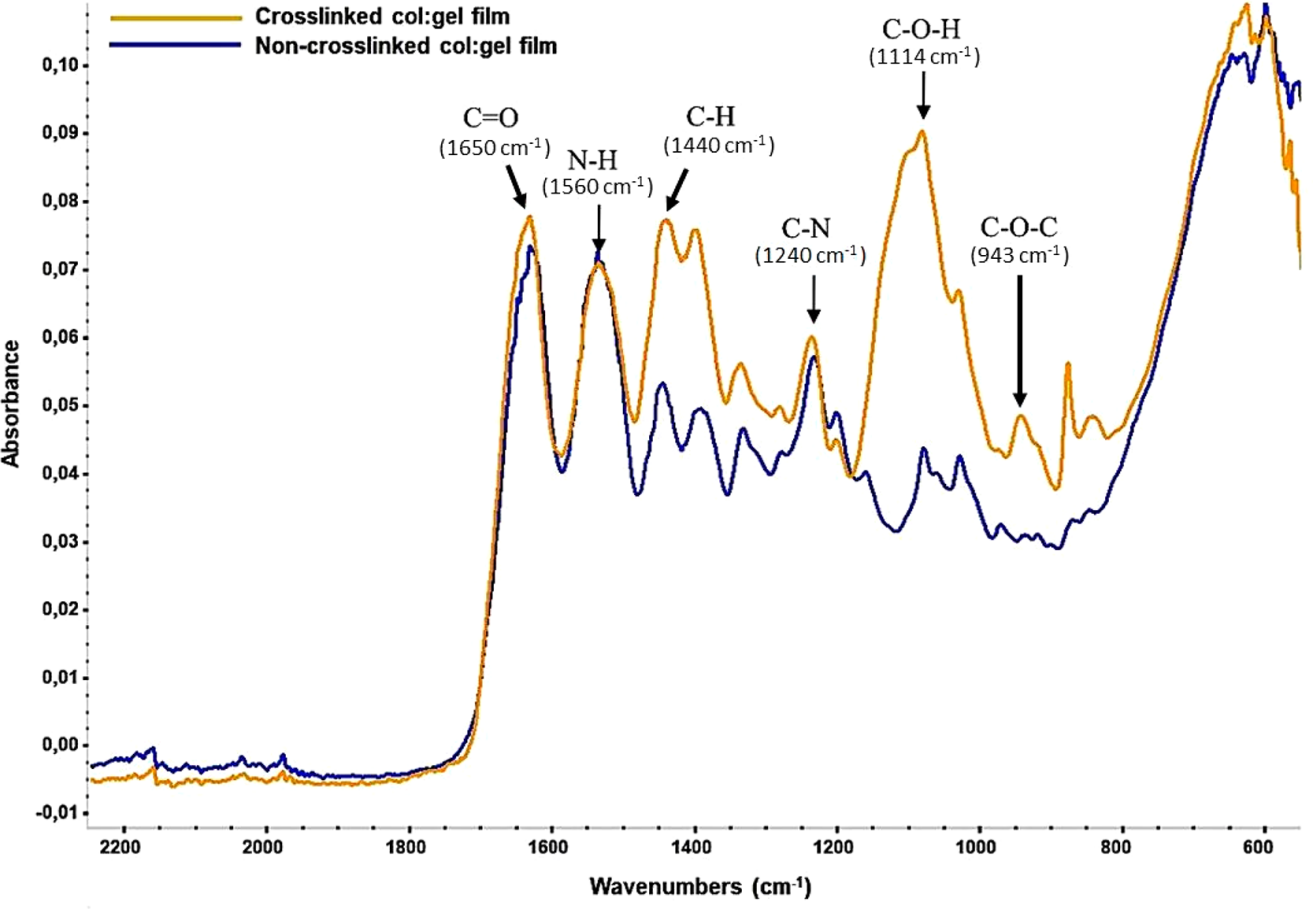
FTIR spectrum of cross-linked and non-cross-linked Col:Gel films.

PEGDE was used as a cross-linker in the biopolymeric mixture to obtain the resultant cross-linked Col:Gel films. The cross-linking reaction occurred between the epoxy groups of PEGDE and the amine and hydroxyl groups of collagen and gelatin chains.^19^ FTIR analysis was performed to identify the functional group of the biopolymeric films and to verify the cross-linking via PEGDE. For both films types, Figure 3 displays the characteristic collagen and gelatin peaks at ∼1650, ∼1560, and ∼1240 cm^–1^ corresponding to C=O stretching vibrations from amide I and N–H bending coupled with C–N stretching vibrations from amide II and amide III, respectively.^34−36^

FTIR results showed that intensity of peaks increased in cross-linked Col:Gel with PEGDE at 1440, 1082, and 943 cm^–1^, compared to the non-cross-linked Col:Gel spectrum (Figure 3). C–H stretching vibrations at 1440 cm^–1^ from CH_2_ increased by incorporation of corresponding groups from PEG main chains and the coupled epoxy rings. In addition, vibration bands corresponding to the C–O–C (at 1082 cm^–1^) and C–O–H (shoulder at 1114 cm^–1^) functional groups dramatically increased for the cross-linked films. The significant improvement in these peaks implies that the epoxy rings of the PEGDE is taking part in the cross-linking reaction.^24,37−39^ Further confirmation of chemical cross-linking was conducted by simple dissolution tests, where the non-cross-linked Col:Gel films immediately dissolves upon immersion into PBS buffer solutions (pH 7.4, 10 mM), but the cross-linked counterparts remain intact for at least 10 days.

**Figure 4:**
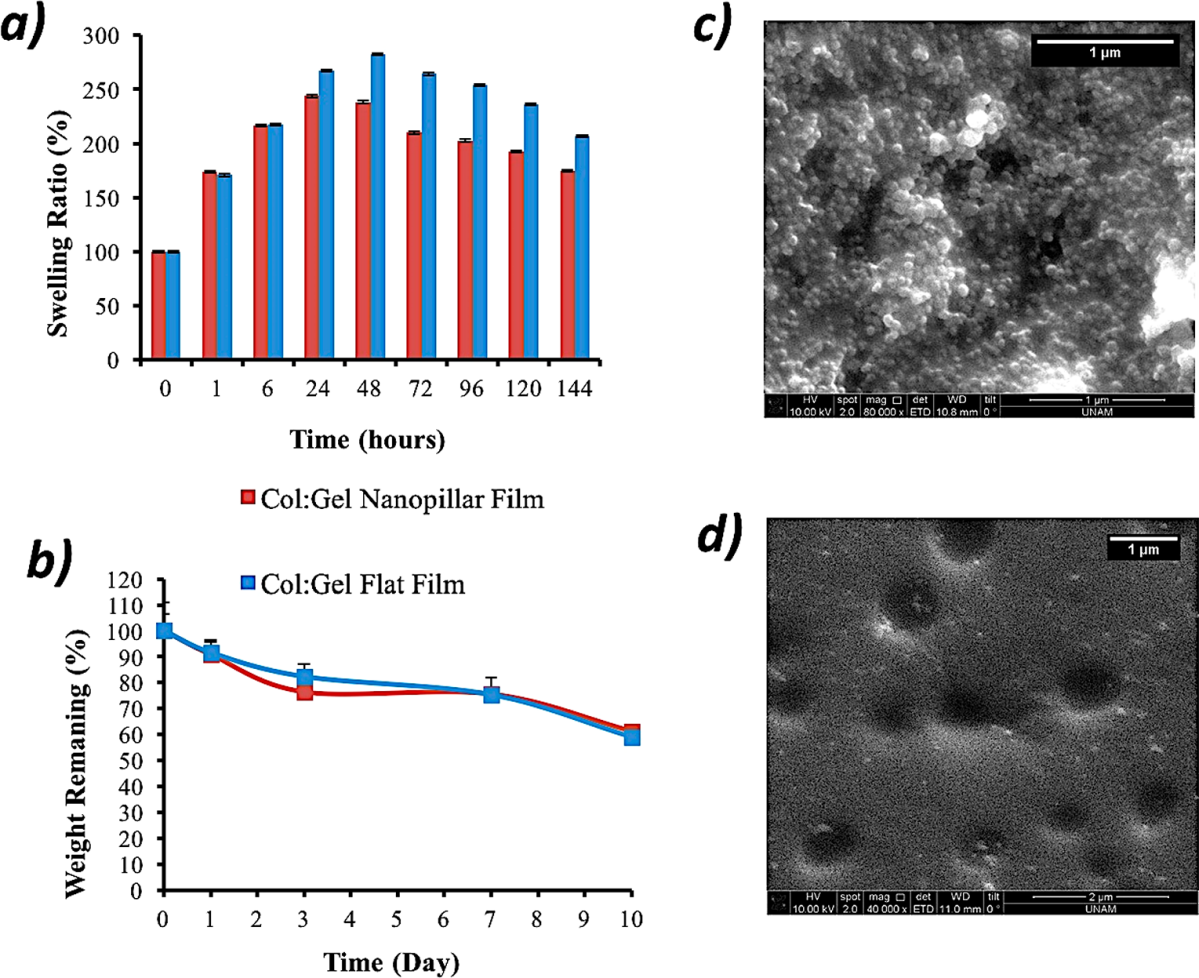
(a) Swelling and (b) degradation profiles of Col:Gel films. (c, d) SEM images of Col:Gel nanopillar film incubated in DMEM for (a) 3 h and (b) 24 h.

The Col:Gel films were further characterized via swelling and degradation tests, and the results are presented in Figure 4. As seen in Figure 4a, swelling of films reached equilibrium after approximately 24 and 48 h for nanopillared and flat films, respectively. This difference can be attributed to the high water absorption capacity of nanopillars stemming from their high surface area. After 24 and 48 h, degradation started to be the dominant phenomenon for nanopillared and flat films, respectively (Figure 4b). Weight remaining of both films was determined as ∼60% after 10 days of incubation (Figure 4b). It is interesting to note that degradation of nanopillared substrates is more extensive especially for the first 3 days, and a similar pattern is also valid for the post equilibrium degradation dominant section of swelling data. It is plausibly again due to the higher surface area of these films causing higher levels of degradation; however since the thickness that the nanodecoration spans is only about 1% of the total film, the difference between the flat counterpart is lost as the degradation test is continued for extended durations.

To examine the persistence of nanotopography, nanopillared films were incubated in DMEM for 3 and 24 h and characterized by SEM (Figure 4c,d). It was observed that the stability of the nanopillar structure was maintained for 3 h in the DMEM medium (Figure 4c), and there were significant losses in the shape and number of the nanopillars due to the swelling/degradation of the film at the end of 24 h (Figure 4d). The swollen craters seen in Figure 4d may have arisen as a result of the local defects present in the honeycomb structure of the AAOM molds.^19^ It was also observed that morphological degradation begins consistently with degradation analysis results and the missing nanopillars in Figure 4d constitutes a fraction of lost weight in Figure 4b at earlier time points.

Mechanical strength of fabricated Col:Gel films was analyzed by tensile test. Films were pulled out at 0.5 mm/min, and load vs elongation values were determined for both film types. The results revealed that nanopillared films have lower ultimate tensile strength values (4.7 ± 6.8 kPa) than the flat counterparts (9.2 ± 1.6 kPa). The reason for this difference can be explained by the stress concentration points of nanostructures. Stress concentration points happen due to the geometrical irregularities that cause an interruption of the stress flow.^40^ Such interruptions can cause earlier disintegration and rupture of the films for the nanostructured substrates yielding poorer mechanical properties.

### 3.3 Cell Culture Studies

**Figure 5:**
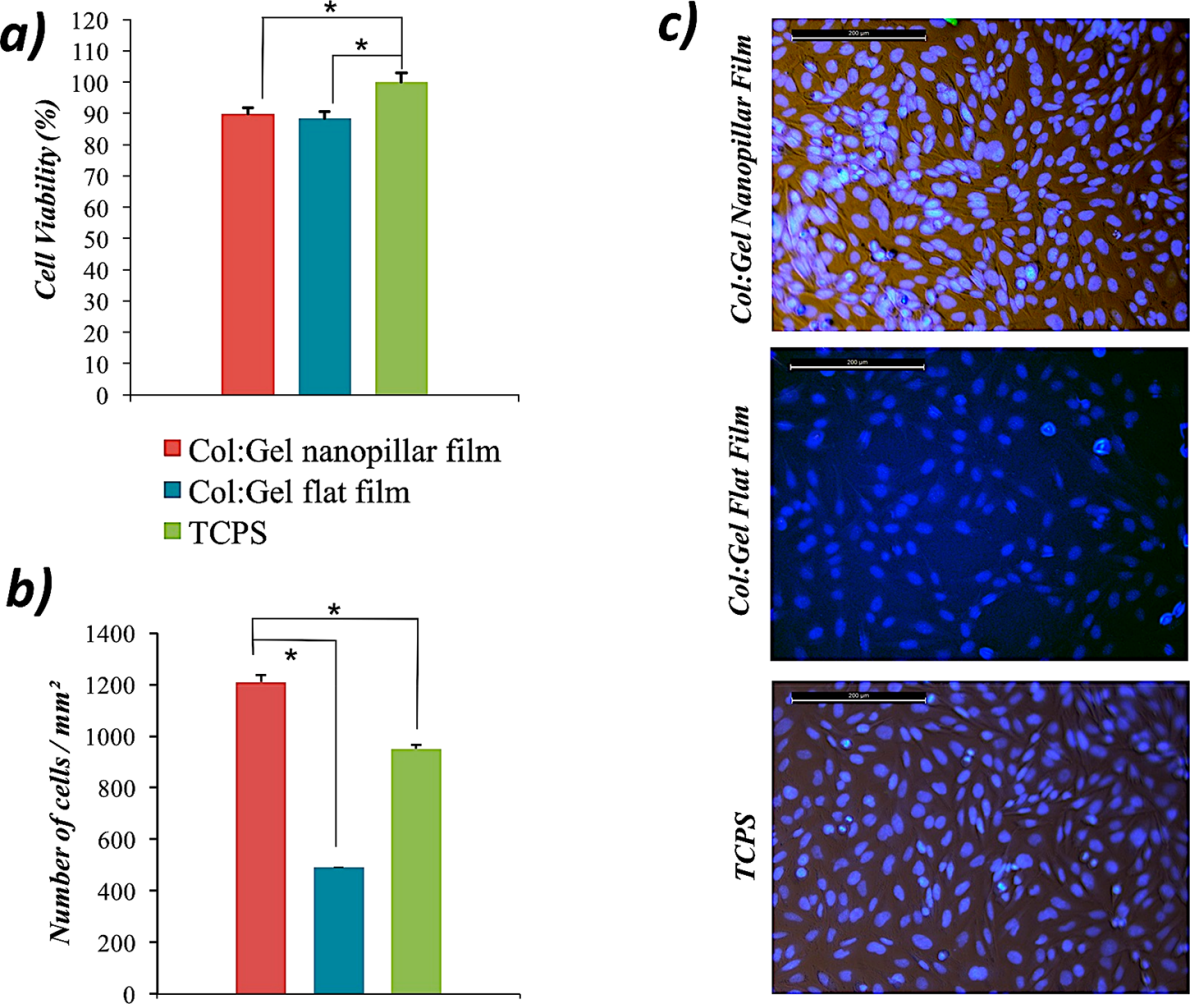
Analysis of (a) cell viability, (b) cell adhesion on nanopillar and flat Col:Gel films and TCP surface. (c) DAPI-stained nuclei of Saos-2 cells on surfaces (scale bar: 200 μm).

The hypothesis of this study was that collagen-based Sharpey’s fiber-inspired nanopillared structure increases adhesion and mineralization of cells as an indicator of early osteogenesis. To confirm this hypothesis, we performed cell culture studies using films seeded with Saos-2 cells. First, the viability of Saos-2 cells on the films was examined via WST-1 analysis (Figure 5a). The absorbance values obtained from the WST-1 analysis change in proportion to cell viability. TCPS (positive control group) surface, which is considered to show 100% cell viability, was chosen as the reference point in viability calculations for the Col:Gel films. Although cells seeded on both nanopillared and flat films have slightly lower viabilities compared to the TCPS surface (*p* < 0.05), the viability values for both substrates are over 90%, and such values can be safely assessed as non-cytotoxic.^41^ The slightly lower viability values of Col:Gel films may be caused due to the potential PEGDE leakage that retain in non-cross-linked native state within the biopolymeric films. Results also showed that there was no statistical significance between the nanopillared and flat Col:Gel groups. To examine the potential of the cell adhesion on the films, the nuclei of the cells were stained with DAPI (Figure 5b,c). As seen in Figures 5 b and c, nanopillared Col:Gel film has the highest number of cells per unit area among the groups.

**Figure 6:**
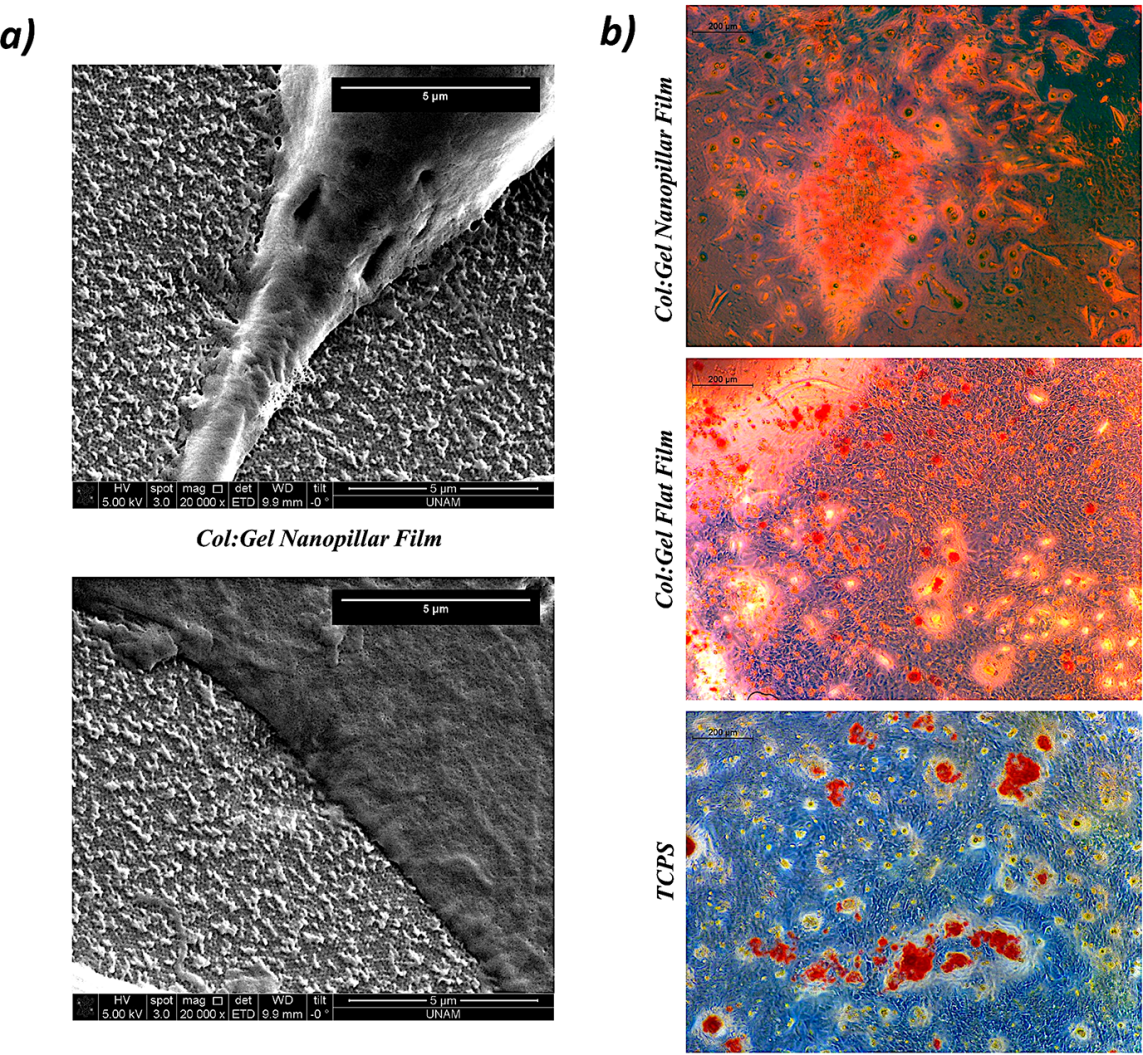
(a) SEM images of Saos-2 cells on the Col:Gel nanopillar film on the 21st day (scale bar: 5 μm). (b) Optical images of mineralized nodules stained via Alizarin Red S at the 21st day of culture (scale bar: 200 μm).

The adhesion of cells on nanopillared films was also confirmed by SEM (Figure 6a). Electron micrographs demonstrated that Saos-2 cells strongly attached to the nanostructured film (Figure 6a). Cellular interactions in the extracellular matrix are mediated through surface receptors called integrins, which collagen and gelatin heavily possess.^8,42^ In addition, nanopillared architecture further supports cell adhesion due to their high surface area (*ca*. ∼ 2 times, by using nanocylinders (pillars) with the features mentioned in section 3.2) and order of magnitude larger roughness values. Surface roughness provokes focal adhesion and serves as a guide for cytoskeleton organization and morphology, and proliferation of cells.^43,44^

As a result, the nanopillared Col:Gel films provide more bioactive sites for sensing cells, which augments formation of focal adhesion and thereby enhance cell adhesion (Figure 6a). Previous studies have demonstrated that rough implant surfaces increase the adsorption of extracellular matrix molecules and cells.^19,45^ The results obtained in this study are in parallel with the literature and prove that nanopillared Col:Gel films provide higher cellular adhesion than both flat counterparts and TCPS control. Note that the chemical content of the biopolymeric films also plays a role in the observed attachment difference. Our previous study where chitosan was the main component of the nanopillared structures lacked this level of cell adhesion difference when compared with the flat counterparts.^19^ The RGD and GFOGER-rich collagen possibly induces drastically different cellular attachment profiles when present in its native nanoscale form. Interestingly, the SEM images also display that the nanopillars continue to exist on the 21st day of cell culture (Figure 6a). These structures disappeared in DMEM after just 1 day when there is no incubated cell in their vicinity (Figure 4d). Nanopillars in the film can retain their stability plausibly due to the adsorption of serum proteins present in the culture media to the substrate surface^46,47^ as well as the adsorption of secretions released by the neighboring cells.

Next, as evidence of osteogenic differentiation, Ca deposition due to mineralization of cells was examined via Alizarin Red S staining. The staining of calcium deposits on each surface is shown in Figure 6b. Although Alizarin Red S staining revealed that both films contained calcium-rich nodules, mineralized regions were more extensive on the nanopillared films (Figure 6b). It is worth noting that flat Col:Gel films had similar levels of nodule formation to that of TCPS control, which implies that nanotopography, when compared to chemical composition, has the dominant role regarding cellular adhesion and osteogenic differentiation on this collagenous biomaterial. Studies have shown that building nanotopography on the surface of biomaterials is advantageous for osteogenic differentiation and promotes osseointegration.^44,48−50^ In the current study, improved surface roughness and area via nanopillar presence not only provokes cellular adhesion but also potentially enhances the adsorption of ECM proteins like collagen,^19^ which paves the way for improved osseointegration as observed in the mineralization results.

Finally, to confirm the presence of deposited minerals on substrate surfaces, Ca and P compositions of the films were specified by Energy Dispersive Analysis of X-rays (EDAX) within the SEM setup and are given in Table 2. Ca and P were determined on all surfaces and the Ca/P values obtained were between 0.97 and 1.35. These values correspond to the form of β-tricalcium phosphate, a bioactive material that increases osteoconductivity.^51,52^

**Table 2:** Elemental Analysis of Mineralized Nodules by EDAX Spectroscopy

	Elemental composition (%)					
Films	C	N	O	P	Ca	Ca/P ratio
Nanopillared Col:Gel	56.61	12.48	25.34	2.44	2.38	0.970
Flat Col:Gel	56.27	11.54	27.16	2.51	2.51	1.002
TCPS	63.28	10.13	28.8	3.84	5.21	1.350

## 4 Conclusions

In this study, inspired by Sharpey’s fibers, which are natural bridges between bone and tooth, nanopillared Col:Gel films were fabricated by the drop-casting method. The Col:Gel films created using AAOM molds exhibited numerous well-organized and uniform nanopillars. Our results demonstrated that nanopillared Col:Gel films with improved surface roughness and surface area values promoted adhesion and osteogenic differentiation of Saos-2 cells when compared to flat films and TCPS controls. We anticipate that nanopillared Col:Gel films that mimic Sharpey’s fibers regarding nanoscale topography, composition, as well as geometrical orientation could impart enhanced osseointegration for dental implants as a potential coating material.
